# Supplementary material for: Evaluating the risk of sleep disorders in subjects with a prior COVID-19 infection
Source: PLoS One. 2024 Oct 17;19(10):e0311929. doi: 10.1371/journal.pone.0311929 (PMC11486372; doi:10.1371/journal.pone.0311929)
Supplement: S1 Table — Abbreviation: COPD, Chronic obstructive pulmonary disease; ADHD, Attention-deficit hyperactivity disorder. (DOCX) [file pone.0311929.s001.docx]

**S1 Table. Standardized differences before and after entropy balancing**

|  | Before Balancing | | | | | | |
| --- | --- | --- | --- | --- | --- | --- | --- |
|  | COVID infection | | | No COVID infection | | | Standardized difference |
| Variable | mean | variance | skewness | mean | variance | skewness |  |
| Age category |  |  |  |  |  |  |  |
| Age 18 to 30 | reference |  |  |  |  |  |  |
| Age 31 to 40 | 0.248 | 0.186 | 1.169 | 0.247 | 0.186 | 1.176 | 0.003 |
| Age 41 to 50 | 0.221 | 0.172 | 1.343 | 0.228 | 0.176 | 1.298 | -0.016 |
| Age 51 to 62 | 0.216 | 0.169 | 1.381 | 0.229 | 0.177 | 1.289 | -0.032 |
| Female | 0.580 | 0.244 | -0.324 | 0.488 | 0.250 | 0.047 | 0.186 |
| Race/Ethnicity |  |  |  |  |  |  |  |
| Non-Hispanic White | reference |  |  |  |  |  |  |
| Non-Hispanic Black | 0.004 | 0.004 | 16.510 | 0.005 | 0.004 | 14.800 | -0.015 |
| Non-Hispanic Asian/Pacific Islander/American Indian | 0.010 | 0.009 | 10.110 | 0.011 | 0.011 | 9.191 | -0.020 |
| Hispanic | 0.024 | 0.023 | 6.221 | 0.021 | 0.021 | 6.680 | 0.020 |
| Unknown | 0.816 | 0.150 | -1.634 | 0.801 | 0.160 | -1.506 | 0.040 |
| Medicaid coverage | 0.083 | 0.076 | 3.022 | 0.117 | 0.103 | 2.385 | -0.123 |
| Depression | 0.183 | 0.150 | 1.636 | 0.123 | 0.108 | 2.296 | 0.156 |
| Anxiety | 0.151 | 0.128 | 1.950 | 0.099 | 0.089 | 2.684 | 0.145 |
| Cognitive disorder | 0.036 | 0.035 | 4.987 | 0.033 | 0.032 | 5.214 | 0.015 |
| Personality disorder | 0.003 | 0.003 | 18.100 | 0.002 | 0.002 | 22.500 | 0.019 |
| Schizophrenia | 0.005 | 0.005 | 13.660 | 0.008 | 0.008 | 11.000 | -0.039 |
| Bipolar | 0.017 | 0.017 | 7.474 | 0.013 | 0.013 | 8.574 | 0.030 |
| Eating disorder | 0.002 | 0.002 | 21.090 | 0.001 | 0.001 | 28.630 | 0.022 |
| ADHD | 0.032 | 0.031 | 5.287 | 0.023 | 0.023 | 6.339 | 0.052 |
| Diabetes | 0.053 | 0.050 | 3.996 | 0.047 | 0.044 | 4.307 | 0.028 |
| Heart failure | 0.002 | 0.002 | 20.860 | 0.002 | 0.002 | 22.800 | 0.008 |
| Cerebrovascular disease | 0.004 | 0.004 | 15.170 | 0.004 | 0.004 | 16.740 | 0.012 |
| Opioid use disorder | 0.012 | 0.012 | 9.105 | 0.009 | 0.009 | 10.400 | 0.025 |
| Thyroid disorder | 0.055 | 0.052 | 3.885 | 0.039 | 0.038 | 4.756 | 0.071 |
| Obesity | 0.170 | 0.141 | 1.755 | 0.115 | 0.102 | 2.407 | 0.146 |
| Asthma/COPD | 0.028 | 0.027 | 5.778 | 0.018 | 0.017 | 7.329 | 0.060 |

**S1 Table. Standardized differences before and after entropy balancing (continued)**

|  | After Balancing | | | | | | |
| --- | --- | --- | --- | --- | --- | --- | --- |
|  | COVID infection | | | No COVID infection | | | Standardized difference |
| Variable | mean | variance | skewness | mean | variance | skewness |  |
| Age category |  |  |  |  |  |  |  |
| Age 18 to 30 | reference |  |  |  |  |  |  |
| Age 31 to 40 | 0.248 | 0.186 | 1.169 | 0.248 | 0.186 | 1.169 | 0.000 |
| Age 41 to 50 | 0.221 | 0.172 | 1.343 | 0.221 | 0.172 | 1.343 | 0.000 |
| Age 51 to 62 | 0.216 | 0.169 | 1.381 | 0.216 | 0.169 | 1.381 | 0.000 |
| Female | 0.580 | 0.244 | -0.324 | 0.580 | 0.244 | -0.323 | 0.000 |
| Race/Ethnicity |  |  |  |  |  |  |  |
| Non-Hispanic White | reference |  |  |  |  |  |  |
| Non-Hispanic Black | 0.004 | 0.004 | 16.510 | 0.004 | 0.004 | 16.500 | 0.000 |
| Non-Hispanic Asian/Pacific Islander/American Indian | 0.010 | 0.009 | 10.110 | 0.010 | 0.009 | 10.100 | 0.000 |
| Hispanic | 0.024 | 0.023 | 6.221 | 0.024 | 0.023 | 6.221 | 0.000 |
| Unknown | 0.816 | 0.150 | -1.634 | 0.816 | 0.150 | -1.633 | 0.000 |
| Medicaid coverage | 0.083 | 0.076 | 3.022 | 0.083 | 0.076 | 3.017 | -0.001 |
| Depression | 0.183 | 0.150 | 1.636 | 0.183 | 0.150 | 1.637 | 0.000 |
| Anxiety | 0.151 | 0.128 | 1.950 | 0.151 | 0.128 | 1.951 | 0.000 |
| Cognitive disorder | 0.036 | 0.035 | 4.987 | 0.036 | 0.035 | 4.986 | 0.000 |
| Personality disorder | 0.003 | 0.003 | 18.100 | 0.003 | 0.003 | 18.100 | 0.000 |
| Schizophrenia | 0.005 | 0.005 | 13.660 | 0.005 | 0.005 | 13.630 | 0.000 |
| Bipolar | 0.017 | 0.017 | 7.474 | 0.017 | 0.017 | 7.473 | 0.000 |
| Eating disorder | 0.002 | 0.002 | 21.090 | 0.002 | 0.002 | 21.090 | 0.000 |
| ADHD | 0.032 | 0.031 | 5.287 | 0.032 | 0.031 | 5.288 | 0.000 |
| Diabetes | 0.053 | 0.050 | 3.996 | 0.053 | 0.050 | 3.996 | 0.000 |
| Heart failure | 0.002 | 0.002 | 20.860 | 0.002 | 0.002 | 20.860 | 0.000 |
| Cerebrovascular disease | 0.004 | 0.004 | 15.170 | 0.004 | 0.004 | 15.170 | 0.000 |
| Opioid use disorder | 0.012 | 0.012 | 9.105 | 0.012 | 0.012 | 9.105 | 0.000 |
| Thyroid disorder | 0.055 | 0.052 | 3.885 | 0.055 | 0.052 | 3.886 | 0.000 |
| Obesity | 0.170 | 0.141 | 1.755 | 0.170 | 0.141 | 1.755 | 0.000 |
| Asthma/COPD | 0.028 | 0.027 | 5.778 | 0.028 | 0.027 | 5.779 | 0.000 |
