# Supplementary material for: Evaluating the risk of sleep disorders in subjects with a prior COVID-19 infection
Source: PLoS One. 2024 Oct 17;19(10):e0311929. doi: 10.1371/journal.pone.0311929 (PMC11486372; doi:10.1371/journal.pone.0311929)
Supplement: Attachment — Submitted filename: Response to Reviewers.docx [file pone.0311929.s002.docx]

April 05, 2024

Dear Dr. Patra and Reviewers,

We appreciate the opportunity to revise our submitted manuscript*, Evaluating the risk of sleep disorders in subjects with a prior COVID-19 infection*. Comments from the reviewers were very helpful in improving the article's focus and we believe the suggestions have made this paper much stronger. Below are the specific reviewer’s comments (in bold) followed by our responses. Newly added parts in the manuscript are highlighted in yellow. Thank you again for taking the time to consider our manuscript.

**Journal Requirements:**

**When submitting your revision, we need you to address these additional requirements.**

**1. Please ensure that your manuscript meets PLOS ONE's style requirements, including those for file naming. The PLOS ONE style templates can be found at** 
[**https://journals.plos.org/plosone/s/file?id=wjVg/PLOSOne_formatting_sample_main_body.pdf**](https://journals.plos.org/plosone/s/file?id=wjVg/PLOSOne_formatting_sample_main_body.pdf) **and** 
[**https://journals.plos.org/plosone/s/file?id=ba62/PLOSOne_formatting_sample_title_authors_affiliations.pdf**](https://journals.plos.org/plosone/s/file?id=ba62/PLOSOne_formatting_sample_title_authors_affiliations.pdf)

Response: We double-checked the style requirements as well as the title, author, affiliations formatting guidelines. Everything is in good shape.

**2. We note that you have indicated that there are restrictions to data sharing for this study. PLOS only allows data to be available upon request if there are legal or ethical restrictions on sharing data publicly. For more information on unacceptable data access restrictions, please see** [**http://journals.plos.org/plosone/s/data-availability#loc-unacceptable-data-access-restrictions**](http://journals.plos.org/plosone/s/data-availability#loc-unacceptable-data-access-restrictions)**.**

**Before we proceed with your manuscript, please address the following prompts:**

a) **If there are ethical or legal restrictions on sharing a de-identified data set, please explain them in detail (e.g., data contain potentially identifying or sensitive patient information, data are owned by a third-party organization, etc.) and who has imposed them (e.g., a Research Ethics Committee or Institutional Review Board, etc.). Please also provide contact information for a data access committee, ethics committee, or other institutional body to which data requests may be sent.**

Response: Data are owned by Utah Department of Health and Human Services, thus requiring approvals from the University of Utah Institutional Review Board and the Utah Resource for Genetic and Epidemiologic Research. We made changes in the Data Availability in the manuscript as follows:

Data cannot be shared publicly due to regulations set by the Utah Department of Health and Human Services, and the Utah Resource for Genetic and Epidemiologic Research (RGE). However, data are available upon approvals from the University of Utah Institutional Review Board ([irb@utah.edu](mailto:irb@utah.edu)) and the Utah Resource for Genetic and Epidemiologic Research (rge@hsc.utah.edu)

**Additional Editor Comments:

This manuscript discusses sleep disorder prior to COVID-19 infection in the UTAH all payers claim database. It is an interesting study, however, there exist several concerns.

1. It will be great to have patient counts beside the patient percentages in tables and tests.**

Response: Thank you for the comment. We have added patient counts in Table 1 so that patient counts in Table 1 can be informative for Tables 2-4.

Additionally, in the first sentence under the Statistical Approach, we have added “counts” as follows:

Mean, standard deviation, counts, and percentages were used to summarize the baseline characteristics of the subjects in 2019.

In Table 1, we have added one more row labeled “Outcome” just above the sleep disorders to clarify that the sleep disorders is the study outcome.

In Tables 2-4, we have corrected the mistakenly reported p-values of 0.00 to <0.01.

**2. In Table 1, most of the % of the patients are same or different by 0.01% except for sleep disorder. Why is that?**

Response: Thank you for the comment. We implemented Entropy Balancing (EB) as a method to balance observed variables between the two groups (i.e. subjects with and without a COVID-19 infection). EB is often used to address confounding in observational studies. After calculating weights for each individual based on EB, statistical insignificance of the variables in Table 1, except for the outcome variable (sleep disorder), indicated that the two groups at baseline (prior to COVID-19 infection) were well balanced. Therefore, potential selection bias was eliminated, and the regression aimed to determine if COVID-19 infection was the primary factor associated with the incidence of a sleep disorder. EB is similar to Inverse Probability Weighting (IPW), but it is known that EB is better than IPW in terms of the accuracy of balancing.

**3. There is a mention of 101% for 51-62 years old, is this a typo?**

Response: Thank you for the question. 101% is correct because the odds ratio of this age group (51-62) compared to the reference group (i.e. 18-30 years old). was 2.01

**4. It will be great to have a flowchart for cohort selection.**

Response: Thank you for the suggestion. We have added a flowchart for cohort selection as Figure 1 in the revision.

Figure 1. Flowchart for the cohort selection

Total number of subjects from 2019-2021

N=3,206,111

Final Number of Subjects

N=413,958

Total number of subjects with continuous enrollment (36 months)

N=1,069,177

Number of subjects with a COVID-19 infection in 2020

N=162,936

Number of subjects with no COVID-19 infection in 2020

N=251,022

Number of subjects excluded due to

Dual eligible any time: N=26,487

Sleep disorder in 2019 and 2020: N=34,994

COVID-19 infection in 2021: N=88,606

Number of adults (18-62 years old) in 2019

N=564,045

**5. In general, table 1 is very confusing. The values in the table look repetitive and it is not outlined how those values have been calculated. Apparently, p values have been used to distinguish both categorical and continuous data, but it is not clear what statistical test has been used. Further, entropy balancing has not been cited.**

Response: The repetitive numbers in Table 1 were a result of balancing the observed variables through Entropy Balancing (EB) process. We aimed to demonstrate the elimination of potential selection bias and confounding associated with the observed variables. This crucial information indicates that selection bias and confounding might be mitigated through balancing the observed variables. In the Statistical Approach section, we described the statistical tests used for comparing the variables in Table 1 as follows:

Following EB, T-tests for continuous variables and Chi-square tests for categorical variables were used to compare the baseline characteristics of the subjects in Table 1.

To provide clarity on EB, we have added two additional references:

Hainmueller J. Entropy Balancing for Causal Effects: A Multivariate Reweighting Method to Produce Balanced Samples in Observational Studies. *Political Analysis*. 2012;20(1):25-46. doi:10.1093/pan/mpr025

Zhao, Qingyuan and Percival, Daniel. Entropy Balancing is Doubly Robust. *Journal of Causal Inference*, vol. 5, no. 1, 2017, pp. 20160010. <https://doi.org/10.1515/jci-2016-0010>

**6. Weighted logistic regression meaning ridge/lasso/something else?**

Response: The Entropy Balancing (EB) method generated weighting values for individuals in the analysis. These weights aim to balance the two groups across the control variables as presented in Table 1. It is necessary to incorporate these weights in the logistic regression, thereby conducting a weighted logistic regression.

**7. Line 125: P-value written as E-value**

Response: Thank you for verifying the information. The E-value is correct. The E-value has been used to assess the magnitude of potential unmeasured confounders for controlled variables, including the primary independent variables. We have added two references for the E-value as follows:

VanderWeele TJ, Ding P. Sensitivity Analysis in Observational Research: Introducing the E-Value. Ann Intern Med. 2017 Aug 15;167(4):268-274. doi: 10.7326/M16-2607. Epub 2017 Jul 11. PMID: 28693043.

Haneuse S, VanderWeele TJ, Arterburn D. Using the E-Value to Assess the Potential Effect of Unmeasured Confounding in Observational Studies. JAMA. 2019;321(6):602–603. doi:10.1001/jama.2018.21554

**Reviewers' comments:

Reviewer's Responses to Questions**

**Please use the space provided to explain your answers to the questions above. You may also include additional comments for the author, including concerns about dual publication, research ethics, or publication ethics. (Please upload your review as an attachment if it exceeds 20,000 characters)**

**Reviewer #1: Your paper "Evaluating the Risk of Sleep Disorders in Subjects with a Prior COVID-19 Infection" offers a noteworthy exploration into the long-term effects of COVID-19 on sleep health. The use of the Utah All Payers Claims Database gives your study a solid foundation, allowing for a comprehensive analysis of post-COVID-19 sleep disorders. Your choice of statistical methods, particularly weighted logistic regression and entropy balancing, effectively addresses potential confounding factors, strengthening the validity of your findings.

However, I would like to point out a couple of areas for potential improvement. The study's geographical focus on Utah might limit the generalizability of your results to broader populations. Additionally, the exclusion of non-prescription medication use and undiagnosed COVID-19 cases could impact the overall picture of sleep disorder prevalence in the post-COVID context. Despite these limitations, your research is an important contribution to understanding the aftermath of COVID-19. It paves the way for further studies, which I hope will expand on your work by incorporating a more diverse set of data and examining additional factors that might influence sleep health after COVID-19 infection. Your work is crucial in highlighting the need for ongoing research into the long-term health consequences of this global pandemic.**

Response: Thank you for the comments. We agree with these points. While this manuscript offers several significant contributions, we recognize the importance of considering two limitations. The current data are specific to Utah, potentially limiting the generalizability of our results to other states. In addition, the data do not include non-prescription medications and undiagnosed COVID-19 cases, as they only provide information covered by insurance companies. We already included these limitations in the Discussion section and hope future studies address these limitations.

Second, the results of the study may not be generalizable to other states as the data used is specific to the state of Utah in the United States. In addition, these findings could not be generalizable to adolescents or the elderly population. Third, prescription medications such as antidepressants and medications for pain, hypertension and asthma that could interfere with sleep were not considered in the study. On the other hand, some antidepressant medications could be prescribed for sleep disorders, but these were not considered to identify patients with sleep disorder. Continuous positive airway pressure (CPAP) therapy is used for patients who have obstructive sleep apnea, but this therapy was not considered to identify sleep disorder in this study.

**Reviewer #2: 1. A careful proofread is recommended to correct any minor typographical errors.**

Response: Thank you for the comment. All authors went through the manuscript to identify and correct any typographical errors. Any changes made during the review were highlighted in the manuscript.

**2. The authors should ensure data availability in line with PLOS's requirements. If there are restrictions, these should be specified.**

Response: Thank you for the comment. Data are owned by Utah Department of Health and Human Services. Thus, approvals from the University of Utah Institutional Review Board and the Utah Resource for Genetic and Epidemiologic Research are required. We made changes in the Data Availability in the manuscript as follows:

Data cannot be shared publicly because of the regulations of Utah Department of Health and Human Services, and the Utah Resource for Genetic and Epidemiologic Research (RGE). Data are available after approvals from the University of Utah Institutional Review Board ([irb@utah.edu](mailto:irb@utah.edu)) and the Utah Resource for Genetic and Epidemiologic Research (rge@hsc.utah.edu)

**3. A more in-depth discussion on the potential pathophysiological mechanisms linking COVID-19 to sleep disorders would enhance the paper's contribution to the existing body of knowledge.**

Response: Thank you for your comment. We agree with the need to expand this section of the discussion. We have added an in-depth discussion highlighting the potential mechanism linking COVID-19 to sleep disorders. We have also added relevant references. Here is the edited paragraph with the added discussion sentences and references highlighted:

The mechanism of the association between COVID-19 infection and sleep disorders is thought to be multifactorial and could encompass factors such as social isolation, ICU stay, direct effect of the viral infection, immunological response, cerebrovascular changes, medication use, among many others [32, 33]. In addition to infecting peripheral tissues linked to the central nervous system (CNS), the SARS-CoV-2 coronavirus also directly enters the brain by permeating the blood-brain barrier (BBB) [34, 35]. This neuroinvasion could potentially affect brain functions, including sleep regulation [35]. Furthermore, hyperinflammatory reactions where the concentrations of C-reactive proteins (CRP) and interleukin-6 (IL-6) are increased may also be implicated in the association between COVID-19 infection and psychiatric symptoms [36]. Specifically, there is a growing body of evidence that COVID-19 infection can lead to elevated levels of inflammatory mediators such as cytokines [37]. Certain cytokines, including IL-6, IL-1α, and tumor necrosis factor (TNF-α), exhibit notable circadian oscillations when they enter the brain, leading to sleep disorders [38, 39]. This relationship between inflammatory mediators and psychiatric symptoms has been previously demonstrated through the link between inflammation and depression [40]. Social isolation also plays a key role in sleep disorders. Sleep disorders were reported to increase during quarantine periods due to COVID-19 infection [41]. During COVID-19 lockdowns, insomnia, poor sleep maintenance, and reduced sleep quality increased for the general population in several countries. This has been attributed to the effect of confinement, worsened by the psychological effects of the disease and increased exposure to artificial lighting from electronic devices [6, 42, 43].

References

34. Burks SM, Rosas-Hernandez H, Alejandro Ramirez-Lee M, Cuevas E, Talpos JC. Can SARS-CoV-2 infect the central nervous system via the olfactory bulb or the blood-brain barrier? Brain Behav Immun. 2021;95:7-14.

35. Granholm AC. Long-Term Effects of SARS-CoV-2 in the Brain: Clinical Consequences and Molecular Mechanisms. J Clin Med. 2023;12(9).

37. Hojyo S, Uchida M, Tanaka K, Hasebe R, Tanaka Y, Murakami M, Hirano T. How COVID-19 induces cytokine storm with high mortality. Inflamm Regen. 2020;40:37.

38. Semyachkina-Glushkovskaya O, Mamedova A, Vinnik V, Klimova M, Saranceva E, Ageev V, et al. Brain Mechanisms of COVID-19-Sleep Disorders. Int J Mol Sci. 2021;22(13).

39. Agorastos A, Hauger RL, Barkauskas DA, Moeller-Bertram T, Clopton PL, Haji U, et al. Circadian rhythmicity, variability and correlation of interleukin-6 levels in plasma and cerebrospinal fluid of healthy men. Psychoneuroendocrinology. 2014;44:71-82.

40. Wohleb ES, Franklin T, Iwata M, Duman RS. Integrating neuroimmune systems in the neurobiology of depression. Nat Rev Neurosci. 2016;17(8):497-511. doi:10.1038/nrn.2016.69

41. Pilcher JJ, Dorsey LL, Galloway SM, Erikson DN. Social Isolation and Sleep: Manifestation During COVID-19 Quarantines. Front Psychol. 2021;12:810763.

42. Limongi F, Siviero P, Trevisan C, Noale M, Catalani F, Ceolin C, et al. Changes in sleep quality and sleep disturbances in the general population from before to during the COVID-19 lockdown: A systematic review and meta-analysis. Front Psychiatry. 2023;14:1166815.

43. Karkala A, Tzinas A, Kotoulas S, Zacharias A, Sourla E, Pataka A. Neuropsychiatric Outcomes and Sleep Dysfunction in COVID-19 Patients: Risk Factors and Mechanisms. Neuroimmunomodulation. 2023;30(1):237-49.

**4. The authors should clarify the severity of COVID-19 cases included in the study and discuss how this factor might influence the outcomes.**

Response: Thank you for the comment. We agree that the severity of COVID-19 infection is critical to be considered. Due to not finding established approaches for defining severity using claims data in the literature, we did not include this part in our initial analysis. However, in this revision, we have categorized the severity of infection into the following categories: No COVID infection, mild COVID infection, and severe COVID infection. Subjects who had ER visits or hospital admissions within 7 days before or after a COVID infection diagnosis were categorized as having a severe COVID infection, while those with a COVID infection but no ER visits or hospital admissions were categorized as having a mild COVID infection.

Based on these definitions, 26.03% of subjects had a mild COVID-19 infection, while 13.33% had a severe COVID-19 infection.

|  | n | % |
| --- | --- | --- |
| No COVID | 251,022 | 60.64 |
| COVID infection | 107,771 | 26.03 |
| Severe COVID infection | 55,165 | 13.33 |

Using these new categories, we ran a weighted logistic regression, which is presented below.

Table S2. Association between the severity of COVID-19 and the risk of sleep disorders

| Covariate | Odds ratio | p-value | 95% confidence interval | |
| --- | --- | --- | --- | --- |
| Severity of COVID-19 |  |  |  |  |
| No COVID infection | Reference |  |  |  |
| Mild COVID infection | 1.26 | <0.01 | 1.19 | 1.33 |
| Severe COVID infection | 1.64 | <0.01 | 1.58 | 1.70 |
| Age |  |  |  |  |
| Age 18 to 30 | Reference |  |  |  |
| Age 31 to 40 | 1.38 | <0.01 | 1.31 | 1.45 |
| Age 41 to 50 | 1.79 | <0.01 | 1.70 | 1.88 |
| Age 51 to 62 | 1.97 | <0.01 | 1.87 | 2.08 |
| Female | 0.81 | <0.01 | 0.78 | 0.84 |
| Race/Ethnicity |  |  |  |  |
| Non-Hispanic White | Reference |  |  |  |
| Non-Hispanic Black | 0.77 | 0.06 | 0.58 | 1.01 |
| Non-Hispanic Asian/Pacific Islander/American Indian | 0.76 | <0.01 | 0.64 | 0.92 |
| Hispanic | 0.82 | <0.01 | 0.72 | 0.92 |
| Unknown | 0.89 | <0.01 | 0.84 | 0.93 |
| Medicaid coverage | 0.91 | 0.01 | 0.85 | 0.98 |
| Elixhauser score | 1.04 | <0.01 | 1.03 | 1.06 |
| Depression | 1.63 | <0.01 | 1.55 | 1.71 |
| Anxiety | 1.58 | <0.01 | 1.51 | 1.66 |
| Cognitive disorder | 1.42 | <0.01 | 1.27 | 1.59 |
| Personality disorder | 1.32 | 0.04 | 1.01 | 1.71 |
| Schizophrenia | 0.90 | 0.26 | 0.76 | 1.08 |
| Bipolar | 1.05 | 0.36 | 0.95 | 1.17 |
| Eating disorder | 1.39 | 0.03 | 1.04 | 1.86 |
| ADHD | 1.21 | <0.01 | 1.07 | 1.36 |
| Diabetes | 0.84 | <0.01 | 0.78 | 0.90 |
| Heart failure | 1.43 | <0.01 | 1.14 | 1.81 |
| Cerebrovascular disease | 1.06 | 0.57 | 0.86 | 1.30 |
| Opioid use disorder | 1.30 | <0.01 | 1.15 | 1.48 |
| Thyroid disorder | 1.10 | 0.01 | 1.03 | 1.18 |
| Obesity | 2.73 | <0.01 | 2.62 | 2.84 |
| Asthma/COPD | 1.26 | <0.01 | 1.15 | 1.38 |

The following parts were added in the Statistical Approach section:

A sensitivity analysis was performed, categorizing COVID-19 infection severity into three groups: no infection, mild COVID-19 infection, and severe COVID-19 infection. Subjects with any emergency room (ER) visits or hospital admissions within 7 days before or after a COVID-19 infection diagnosis were categorized as having a severe COVID-19 infection, while those without such visits or hospital admissions were categorized as having a mild COVID-19 infection.

The following sentences have been added in the Results section:

The sensitivity analysis with categories of COVID-19 infection severity showed that subjects with severe COVID-19 infection had a higher risk of experiencing sleep disorders than those with mild or no COVID-19 infection. Compared to subjects with no COVID-19 infection, those with mild COVID-19 infection were 26% more likely to have sleep disorders in the following year (OR=1.26, p<0.01), while subjects with severe COVID-19 infection had a 64% higher likelihood of having sleep disorders (OR=1.64, p<0.01). To compare those with mild infection to those with severe infection, we conducted a Wald test resulting in a statistically significant difference (F-value=348, p<0.01) between the two groups. This indicates that those with severe infection experienced higher rates of sleep disorders than those with mild infection (S2 Table).

The new regression table has been added as S2 Table.

**5. The paper would benefit from a limitations section that discusses the potential for unmeasured confounding, given the observational study design.**

Response: Thank you for the comment. We agree with this point. Despite our efforts to balance the observed confounders using Entropy Balancing, the results may still be affected by unmeasured confounders. We have added the following limitation in the Discussion section:

While we controlled for observed variables that were potentially associated with the cause of sleep disorders, unmeasured confounders could potentially influence the results of the study, given the observational study design.

**6. In the results section, the authors should discuss the practical implications of their findings for healthcare providers and public health policy.**

Response: Thank you for the comment. We have discussed practical implications of our findings for healthcare providers and policymakers. We added the following highlighted sentences and references:

Sleep disorders are recognized as important public health and economic concerns requiring prompt attention [45]. Thus, our study’s findings have various implications for healthcare providers and policymakers. This study contributes to the existing body of literature available to healthcare providers, highlighting the potential impact of COVID-19 infection on sleep. It may prompt providers to consider incorporating sleep-related questions into patient assessments, particularly considering that symptoms of sleep disorders may manifest in different ways and could be difficult to link specifically to the infection, especially in the absence of definitive diagnostics [46]. Additionally, healthcare providers can offer psychological support to patients who may feel unheard when complaining about their symptoms [46]. Healthcare providers could be instrumental in managing sleep disturbances, as well as in assessing patients’ progress through sleep pattern tracking and assisting patients in setting realistic goals for their recovery [47]. Cognitive behavioral therapy and progressive muscle relaxation are intervention that have been used to treat sleep disorders in patients with COVID-19 infection [48, 49]. In a randomized controlled trial, Liu et al reported that PMR reduced anxiety levels and improved sleep quality after 5 days [49]. The findings of this study were inconsistent with that of another study by Masih et al which showed no difference between intervention groups [50]. Other alternative therapies include short-term benzodiazepines and hypnosis [51].

Moreover, our study generates relevant scientific evidence that will better equip policymakers in their decision-making process [52]. Policies that could stem from our findings encompass launching public health campaigns aimed at raising awareness about the potential impact of COVID-19 on sleep and the importance of seeking timely medical attention for sleep-related issues. Other policies include establishing integrated care models that would ensure collaborative care and allocating funding for research and surveillance to sustain continued research into the relationship between COVID-19 and sleep disorders.

References

46. Landhuis EW. How Primary Care Physicians Can Recognize and Treat Long COVID. JAMA. 2023;329(20):1727-9.

47. Greenhalgh T, Sivan M, Delaney B, Evans R, Milne R. Long covid-an update for primary care. BMJ. 2022;378:e072117.

52. Gombos K, Herczeg R, Eross B, Kovacs SZ, Uzzoli A, Nagy T, et al. Translating Scientific Knowledge to Government Decision Makers Has Crucial Importance in the Management of the COVID-19 Pandemic. Popul Health Manag. 2021;24(1):35-45.
