# Supplementary material for: Evaluating the risk of sleep disorders in subjects with a prior COVID-19 infection
Source: PLoS One. 2024 Oct 17;19(10):e0311929. doi: 10.1371/journal.pone.0311929 (PMC11486372; doi:10.1371/journal.pone.0311929)
Supplement: Attachment — Submitted filename: Response to Reviewers.docx [file pone.0311929.s003.docx]

May 25, 2024

Dear Dr. Patra and Reviewers,

We appreciate the opportunity to revise our manuscript*, Evaluating the risk of sleep disorders in subjects with a prior COVID-19 infection*. Below are the specific reviewer’s comments (in bold) followed by our responses. Newly added parts in the manuscript are highlighted in yellow. Thank you again for taking the time to consider our manuscript.

**Reviewer #2: All the comments are well addressed. The manuscript is now in publishable form. Other typos have also been addressed.**

Response: Thank you for taking the time to review the revised manuscript.

**Reviewer #3: The authors studied the impact of covid on sleep disorders using the Utah all payers claims database. The results of the study can provide valuable insights to our understanding of covid which can benefit clinicians, public health workers, and patients. The study is well-designed, although I believe the analysis should be improved before the manuscript is ready for publication.**

**Elixhauser comorbidity index is for predicting in-hospital mortality and resource use. I’d assume most covid or sleep disorders patients would not require hospitalization, so can the authors justify why the included the Eixhauser comorbidity index? Also, some dx included in deriving the Eixhauser comorbidity index overlap with some covariates the authors included in the models (e.g., depression, obesity), so these variables were somehow adjusted twice in the models.**

Response: Thank you for your comment. Studies have indicated that sleep disorders, including sleep apnea and insomnia, may be associated with an increased risk of mortality (Zolfaghari et al., 2024; Cappuccio et al., 2010; Lin et al., 2023). Additionally, the Centers for Disease Control and Prevention reported 848,943 deaths due to COVID-19 in the United States between 2020 and 2021 (source: <https://www.cdc.gov/nchs/covid19/mortality-overview.htm>). Therefore, we deemed it important to control for a comorbidity index such as Elixhauser.

The Elixhauser comorbidity index assigns weights to each condition and then sums these weights for each subject. Because this index considers multiple diseases **dependently**, we believe that controlling for individual comorbidities, even though they are included in the Elixhauser index calculation, would be beneficial for enhancing the overall goodness of fit of the regression.

In response to the comment above, we conducted a regression excluding the Elixhauser comorbidity index and only controlled for the individual comorbidities. The results, including odds ratios and p-values of the control variables, remained consistent with and without the inclusion of the Elixhauser index variable. Specifically, the odds ratio of the independent variable (i.e., COVID infection: OR=1.53, p<0.01 in Table 2) remained unchanged in both regressions. Subsequently, we updated the previous results with those from the regression excluding the Elixhauser index variable and made corresponding revisions to the Results section.

References

Zolfaghari S, Keil A, Pelletier A, Postuma RB. Sleep disorders and mortality: A prospective study in the Canadian longitudinal study on aging. Sleep Med. 2024 Feb;114:128-136. doi: 10.1016/j.sleep.2023.12.023

Cappuccio FP, D'Elia L, Strazzullo P, Miller MA. Sleep duration and all-cause mortality: a systematic review and meta-analysis of prospective studies. Sleep. 2010 May;33(5):585-92. doi: 10.1093/sleep/33.5.585.

Lin Y, Wu Y, Lin Q, et al. Objective Sleep Duration and All-Cause Mortality Among People With Obstructive Sleep Apnea. JAMA Netw Open. 2023;6(12):e2346085. doi:10.1001/jamanetworkopen.2023.46085

Centers for Disease Control and Prevention. COVID-19 Mortality Overview. Access on May 22, 2024 at <https://www.cdc.gov/nchs/covid19/mortality-overview.htm>.

**Entropy balancing is for casual inference. Although from the study design I can see the authors wanted to do some causal inference (dropped people with sleep disorders dx before 2021), but then they why did you balance the covariates and ALSO control for them in the logistic models?**

Response: Thank you for the comment. Controlling the covariates that were used in the Entropy Balancing enhances the robustness of the estimation through doubly robust estimation. If either the Entropy Balancing model or the main regression model is correctly specified, the estimation remains consistent. This approach offers increased protection against model misspecification and enhances the reliability of causal inference in the study.

**I don’t see the point of separate models for private and medicaid beneficiaries (tables 3 and 4). The full model (table 2) already included insurance type (medicaid coverage indicator) as a main effect, which means you were assuming that the effects of all other covariates would be the same for medicaid and private beneficiaries, which seems to be a valid assumption since the two subgroup models (tables 3 and 4) had similar results. If you expect the effects of covariates to vary by insurance type, you should include interaction terms between medicaid coverage and all other covariates in the full model (table 2).**

Response: Thank you for the comment. We wanted to explore potential differences in the main effect, specifically the odds ratio of the COVID-19 infection variable, as we considered subgroups based on insurance type (Medicaid only vs. private insurance only). Individuals covered by Medicaid may experience a more pronounced negative impact (OR=1.67, p<0.01) on sleep problems due to COVID-19 infection compared to those covered by private insurance (OR=1.51, p<0.01), although direct comparisons of odds ratios are not feasible (i.e. two regressions with two different number of subjects).

Having interaction terms between Medicaid and all other covariates in the full model has to control over 25 additional variables in Table 2. Also, comparing any differences in Medicaid vs. private insurance is not the main purpose of the paper. Thus, we would like to keep Table 2 as it is. Now, we removed Table 3 (i.e. regression results for those covered by private insurance) and 4 (i.e. regression results of those covered by Medicaid). S2 Table (Association between COVID-19 severity and risk of sleep disorders) has been moved to Table 3. In this model, we removed the Elixhauser index variable and reran the regression. Relevant texts were revised in the paper.

**Discussion — It’s very hard to follow some of the literature summaries in the discussion section. E.g, in the second paragraph, the authors started of the paragraph saying their results were consistent with what had been published. But later in the paragraph they mentioned more studies reported “however, sleep disorders … as a long-term effect associated with prior COVID-19 infection”. From the summaries of the studies, It seems to me that the first few studies before the transition sentence (however…) were cross-sectional studies, and studies after the transition sentence were longitudinal studies looking at long term effects. But the starting sentences did not send off this message and were somehow misleading**.

Response: Thank you for the comment. In the paragraph, we first tried to present studies that reported incidences of sleep disorders **during** the COVID-19 pandemic, followed by studies that reported sleep disorders as a long-term effect associated with prior COVID-19 infection. To address your concerns, we have restructured the flow of the paragraph to make it less confusing and easy to understand. We also changed “however” to “moreover” as the transition word since the studies reported incidences of sleep disorder both during and after COVID infection.

**Some references were missing — authors mentioned xxx et al. did yyy, but there was no citation at the end. I highly recommend the authors carefully go through the draft again before resubmission. There are some broken sentences.**

Response: The authors went through the manuscript and the references to make sure.
