# Supplementary material for: Evaluating the risk of sleep disorders in subjects with a prior COVID-19 infection
Source: PLoS One. 2024 Oct 17;19(10):e0311929. doi: 10.1371/journal.pone.0311929 (PMC11486372; doi:10.1371/journal.pone.0311929)
Supplement: Attachment — Submitted filename: Response to Reviewers.docx [file pone.0311929.s004.docx]

September 03, 2024

Dear Dr. Patra and Reviewers,

We appreciate the opportunity to revise our manuscript*, Evaluating the risk of sleep disorders in subjects with a prior COVID-19 infection*. Below are the specific reviewer’s comments (in bold) followed by our responses. Newly added parts in the manuscript are highlighted in yellow. Thank you again for taking the time to consider our manuscript.

**Reviewer #3: : I appreciate the authors taking time to address all my comments. For the controlling for covariates in regression models after Entrophy Balancing part, I'd recommend the authors mention this in the "statistical approach" section that the weighted regression model also controlled for covariates, and cite a few published papers that have done so.**

Response: Thank you for the comment. We added the following sentence in the Statistical Approach section with two references:

The weighted regression model controlled for the covariates that were included in the EB.

References

Sudduth, J. D., et al. Preoperative opioid use and its association with postoperative complications. Journal of Substance Use. 2024: 1–7. https://doi.org/10.1080/14659891.2024.2351016.

Ricci C., et al. Appendiceal goblet cell carcinoma has marginal advantages from perioperative chemotherapy: a population-based study with an entropy balancing analysis. Langenbecks Arch Surg. 2023 Jan 25;408(1):65. doi: 10.1007/s00423-023-02791-x.

**One other minor comment: P3, line 49, “sleep disorders may be a result of” — there was an additional “as” before “a result of” in the manuscript.**

Response: Thank you for pointing it out. It was an error, and we have corrected it as follows:

“Sleep disorders may be a result of various factors ranging from stressful life events…”

**Reviewer #4: The paper studies the impact of covid -19 on sleep disorders. This is done by building a weighted logistic regression model taking patient characteristics including the occurrence of COVID-19 as independent variables and occurrence of sleep disorders as dependent variable.**

**1. The AUC/ROC for logistic regression is not included.**

**Response:** Thank you for the comment. The area under the ROC curve (AUC) for both Tables 2 and 3 is 0.72, indicating that there is a 72% chance that the model is able to distinguish between positive cases and negative cases.

ROC for Table 2:

ROC for Table 3:

According to Hosmer & Lemeshow (Applied logistic regression, 2013), our AUC/ROC=0.72 offers acceptable discrimination.

0.5 ≤ no discrimination

0.5-0.7 = poor discrimination

0.7-0.8 = Acceptable discrimination

0.8-0.9= Excellent discrimination

>0.9 = Outstanding discrimination

We added the following sentences in the Statistical Approach section:

The area under the receiver operating characteristic (ROC) curve (AUC) was used to measure the performance of the logistic regression models.

We added the following result in the Results section:

The AUC following the logistic regressions were 0.72 for both Tables 2 and 3, indicating that there is a 72% chance of the models distinguishing between positive and negative cases.

Reference

Hosmer Jr., D.W., Lemeshow, S. and Sturdivant, R.X. (2013) Applied Logistic Regression. 3rd Edition, John Wiley & Sons, Hoboken, NJ.

**2. There is no description of the weighting scheme used by the logistic regression model.**

**Response:** Thank you for the comment. We added the following parts in the Statistical Approach section:

The EB approach for a binary outcome is based on a maximum entropy reweighting scheme that assigns weights to each subject so that the control group (i.e. no COVID infection group in this study) is reweighted to match the controlled covariate moments (i.e. mean, standard deviation, and variance in this study) in the treatment group (i.e. COVID infection group in this study). Therefore, EB ensures that the two groups being compared are similar enough by reweighting the patient characteristics (i.e. covariates)

We have added three references for these parts:

Zhao, Qingyuan and Percival, Daniel. "Entropy Balancing is Doubly Robust" *Journal of Causal Inference*, vol. 5, no. 1, 2017, pp. 20160010. <https://doi.org/10.1515/jci-2016-0010>

Hainmueller, J., & Xu, Y. (2013). ebalance: A Stata Package for Entropy Balancing. *Journal of Statistical Software*, *54*(7), 1–18. https://doi.org/10.18637/jss.v054.i07

Markoulidakis, A., Taiyari, K., Holmans, P. *et al.* A tutorial comparing different covariate balancing methods with an application evaluating the causal effects of substance use treatment programs for adolescents. *Health Serv Outcomes Res Method* **23**, 115–148 (2023). https://doi.org/10.1007/s10742-022-00280-0

We appreciate your time to review the manuscript!
